# Balance Enhancement in Older Adults: Is Functional-Task Training Better Than Resistance Training in Enhancing Balance in Older Adults?

**DOI:** 10.7759/cureus.19364

**Published:** 2021-11-08

**Authors:** Rafi Mohammed, SD Shahanawaz, Pallavi Dangat, Gaurav Bhatnagar, Shyam Jungade

**Affiliations:** 1 Community Health Physiotherapy, Maharashtra Institute of Physiotherapy, Latur, IND; 2 Neurological Physiotherapy, College of Applied Medical Sciences, University of Hail, Hail, SAU; 3 Neurological Physiotherapy, Maharashtra Institute of Physiotherapy, Latur, IND; 4 Orthopedic Physiotherapy, Maharashtra Institute of Physiotherapy, Latur, IND

**Keywords:** resistance, training, functional-task, older adults, balance

## Abstract

Background and purpose

The effects of various exercise training programs on balance in older adults are well established. This study aimed to compare the effect of functional-task training with resistance training in improving balance performance in older adults.

Methods

A total of 100 community-dwelling older adults aged 65 years and above were randomly allocated into two groups: functional-task training (FTT) group and resistance training (RT) group. The FTT group (n = 50) performed functional task exercises and the RT group (n = 50) performed resistance exercises three times a week for 12 weeks. Balance was evaluated before and after the trial using the Berg Balance Scale (BBS) and the Timed Up and Go (TUG) test.

Results

A total of 87 subjects who completed the study were analyzed. Both the groups showed a significant change in BBS and TUG test (p < 0.05) from baseline to 12 weeks. However, post-intervention analysis between the groups showed a significant difference in both the BBS and TUG test (p < 0.05), i.e. improvement in the FTT group was better than the RE group at the end of training.

Conclusion

Both the FTT and RT were effective in improving balance. However, the improvement achieved by the FTT group was better than the RT group.

## Introduction

Adults aged 65 years and above represent one of the fastest-growing segments of Indian as well as of the international population. As a result of socio-economic development and enhanced medical services, there is an apparent increase in the number of older adults during the past few decades. By the year 2050, the number of older adults is expected to cross 300 million in India [1].

Aging involves a progressive, gradual, and natural deterioration of various physiological functions including balance [2-4]. Balance is a generic term that describes the dynamics of body posture to prevent falls. It is related to inertial forces and the inertial characteristics of the body and its segments [5]. Balance in older people deserves special attention because of its importance in functional mobility and safety. With the increase in the aging population and increased life expectancy, maintaining functional status is becoming very important. Balance discrepancy is one of the major risk factors for falls among older adults. A high correlation was found between balance deficit and fall incidence [6]. A fall may result in injury, disability, or loss of life. The other consequences of fall may be the development of fear of future fall that would lead to a decline in mobility and functional independence [7]. Inability to maintain balance is not only a result of disease but also a normal aging process [8]. Age-related changes in the three sensory systems, i.e. vestibular [9], visual [10], and somatosensory [11], interfere with the ability to balance effectively. Other factors like decreased lower-body strength, coordination, and flexibility also contribute to a decrease in balance ability [12]. These changes consequently affect reaction time in the elderly and increase balance-related issues. Activities such as reaching or bending involve a shift of the center of gravity (COG) of the body within the base of support (BOS). Whenever the COG moves outside the BOS, balance is maintained by execution of an automatic movement strategy either by bringing back the COG within the BOS or a new BOS is established by suggesting a step strategy. When a proper movement strategy execution does not occur, the person may fall [13].

Balance is generally disturbed when a simultaneous secondary task is performed. Older adults are unable to sustain balance during activities when their attention is divided between tasks [14]. It is not only in balance-impaired elderly, but healthy older adults also show reduced physical performance while performing attention-demanding tasks, which increase the risk of falls even more [6]. The circumstances like the simultaneous performance of a secondary task arise more often in everyday situations. Therefore, specific exercise training is needed to improve the ability to perform a secondary task simultaneously without losing balance. Functional-task training can be used for this purpose.

Traditionally, resistance training was used as an exercise intervention to treat balance deficits in older adults. However, the effects of resistance exercises are inadequate to enhance balance and other functional activities. Furthermore, resistance exercises when administered alone, without another component, were reported unproductive [15]. Resistance exercises mostly tend to target one or more muscle groups to improve their function. Functional-task training rather focuses on the level of activity by improving strength and altering postural strategies concerning environmental demands. Performance of functional tasks requires well-organized functional movements and task-oriented balance abilities and integrates several muscles and joints moving in various planes. Functional-task training includes balance and coordinated movement strategies together with muscular control activities required to live independently in the dynamic environment [16]. In this context, an intervention consisting of functional-task training may yield enhanced results than other forms of training when administered to older individuals for improving balance and preventing falls. Therefore, the main purpose of this study was to explicate whether functional-task training is better than resistance training in improving balance in Indian community-dwelling older adults.

## Materials and methods

Study design and participants

This is a single-blinded, comparative experimental design. A total of 100 community-dwelling elderly were recruited from recreational parks and religious places. Both males and females aged ≥65 years, who were apparently healthy, without cognitive impairment (Mini-Mental State Examination (MMSE) ≥ 24) [17], able to lift 1.5 kg weight from the floor and stand up, able to walk with or without an assistive device, and without the assistance of another person were included in the study. Subjects who had neurological diseases, low or high uncontrolled blood pressure, uncontrolled cardiovascular and respiratory condition, musculoskeletal diseases or surgeries, were on medication that could affect their balance, and subjects already participating in a physical activity program were excluded from the study. The study was approved by two ethical committees: (i) Department Research Committee (DRC) of Maharaj Vinayak Global University, Jaipur, India, and (ii) Board of Research Studies (BORS) of the Maharashtra Institute of Physiotherapy, Latur, India.

The participants were given a complete explanation of the study before obtaining written informed consent. Each participant was randomly assigned to one of the two groups, functional-task training (FTT) group or resistance training (RT) group, by an independent researcher using cards in unmarked envelopes.

Intervention

The two groups of study subjects were assigned to participate in either FTT or RT. The FTT group performed the exercises that mimic functional activities, while the RT group performed resistance exercises that mainly focus on lower extremity muscle performance (details of the exercise training programs can be found in the Appendix). Exercises were performed thrice a week for a total of 12 weeks. The one-hour session of the exercise was divided into a warm-up period comprising aerobic exercises for 10 minutes, core exercises for 40 minutes, and a cool-down period comprising flexibility exercises for 10 minutes. Borg perceived exertion scale (Borg CR10) was used to set the exercise intensity [18]. For both the training programs, the maximum intensity used was "5," i.e. "severe" level of exertion on Borg CR10. The exercise load was increased by using more weight, repetitions, duration, or speed of exercise till the exercise intensity became "severe" on Borg CR10.

Outcome measures

Berg Balance Scale

The Berg Balance Scale (BBS) is generally considered to be the gold standard and is the widely used test for measuring static and dynamic balance in elderly people [19]. It consists of a set of 14 simple and common balance-oriented tasks. For example, single-leg stand, standing from sitting position, reaching, tandem walking, 360 degrees turning, etc. It is measured on an ordinal scale of 0 to 4 points, where 0 signifies a failure to perform the task and 4 signifies performing the task independently. Thus, a score of 56 suggests total independence.

Timed Up and Go Test

The Timed Up and Go (TUG) test is used to assess balance as well as mobility in elderly people [20]. It measures the time that an individual takes to rise from a chair, walk a distance of 3 meters, turn around, and walk back to sit again on the chair. Before testing, the subjects are given a practice trial that is not timed.

Statistical analysis

Unpaired t-test or chi-square tests were used to compare the general characteristics and baseline data between the groups. Paired t-test was used to compare the pre- and post-intervention data of the groups. Post-intervention data between the groups were compared using the unpaired t-test. The significance level for all the analyses was set at ≤0.05. Effect size (Cohen's d) between the groups was calculated by dividing the mean difference by the pooled standard deviations. An effect size of 0.20-0.49 is considered small, while 0.50-0.79 is moderate, and an effect size of 0.80 and above is large [21]. GraphPad Prism 9.1.0 (GraphPad Software, San Diego, CA) was used to perform all analyses.

## Results

Of 100 participants who were enrolled, 87 participants completed the study (46 in the FTT and 41 in the RE group). The age of the participants was between 65 and 83 years, the mean being 73.63 ± 4.62 years. There were 53 men 47 women. Table 1 presents the demographic characteristics and baseline data of the variables. The groups show homogeneity with regard to age, sex, body height, body weight, and BMI.

**Table 1:** Baseline data of the study participants. FTT: functional-task training; RT: resistance training; BMI: body mass index; BBS: Berg Balance Scale; TUG: Timed Up and Go; * unpaired t-test; † chi-square test.

	FTT group (n = 46)	RT group (n = 41)	P
Age	73.41 ± 4.24	72.76 ± 4.16	0.46^*^
Males	25	22	0.94^†^
Females	21	19	
Height (meters)	162.15 ± 7.59	164.24 ± 8.67	0.23^*^
Weight (kg)	62.57 ± 8.91	62.54 ± 8.55	0.98^*^
BMI (kg/m^2^)	23.71 ± 2.35	23.14 ± 2.27	0.25^*^
BBS	40.57 ± 4.73	40.90 ± 4.57	0.73^*^
TUG (seconds)	11.04 ± 1.44	10.85 ± 1.64	0.56^*^

Results of the BBS and TUG scores of subjects are summarized in Table 2. BBS scores of both the FTT and RT groups improved significantly after the training (p < 0.05). For the TUG test, a significant decrease in the time of completion of the test was noted in both the groups after the training (p < 0.05). However, the FTT group improved significantly better than the RT group, i.e. results of the unpaired t-test show that there was a statistically significant difference (p < 0.05) between the groups in respect to both BBS and TUG at the end of the training (Table 3). The effect size was small and moderate for BBS and TUG, respectively.

**Table 2:** Comparison of the balance measures between pre- and post-intervention. FTT: functional-task training; RT: resistance training; BBS: Berg Balance Scale; TUG: Timed Up and Go; * paired t-test.

	Pre	Post	P-value*	% change
FTT (n = 46)				
BBS	40.57 ± 4.73	44.63 ± 5.11	<0.0001	10.03
TUG	11.04 ± 1.44	9.39 ± 0.98	<0.0001	14.94
RE (n = 41)				
BBS	40.90 ± 4.57	42.12 ± 5.13	<0.0001	2.98
TUG	10.85 ± 1.64	10.05 ± 1.45	<0.0001	7.37

**Table 3:** Comparison of post-intervention measures between the groups. FTT: functional-task training; RT: resistance training; BBS: Berg Balance Scale; TUG: Timed Up and Go; * unpaired t-test; § Cohen’s d.

	FTT group (n = 46)	RT group (n = 41)	P*	Effect size^§^
BBS	44.63 ± 5.11	42.12 ± 5.13	0.025	0.49
TUG	9.39 ± 0.98	10.05 ± 1.45	0.014	0.53

## Discussion

Both the functional-task training and the resistance training groups benefited from the training programs with a significant improvement in balance. Post-intervention group analyses showed that the participants who received the functional-task training improved better than the resistance training group, i.e. there was a significant difference in both BBS and TUG test scores between the groups after the completion of training. The main reason for the better improvement in balance performance in the FTT group was that the inclusion of the functional tasks they put into practice. These tasks are associated with activities that can encourage participants to turn, bend, and reach the limits of stability and thus offering further vestibular stimulation. The increase in speed and repetition of movements during the progression of an exercise might have improved flexibility, strength, endurance, and reaction time in the FTT group. This observation is supported by the previous studies that used multi-task balance training programs [22,23]. In a study that incorporated functional task exercises, the effects of the training were sustained for the long term than the gains achieved by strength training [16].

Although strength is an important component of balance ability in the prevention of falls, strengthening exercise without balance training may not be helpful in preventing falls [15,24]. Moreover, the effects of strength training on the functional performance of the elderly were not well established [25,26]. While it was assumed that resistance exercises are not an effective intervention for the elderly population, to our surprise, the group that performed resistance training in our study also improved significantly, although not as good as the FTT group. This improvement may be because the resistance training helped to improve strength in the lower limbs, which added an indirect constructive effect on balance [27]. However, task-specific multi-component exercises, turning, and reaching were not the integral components of the resistance training program. These differences in the training program might have contributed to the different results. Various studies reported that enhancement in balance requires a functional, task-oriented, or specific balance training program. According to a systematic review, exercise programs that are helpful in reducing falls incorporate a mixture of various exercise programs, typically task-oriented functional exercises, strength training, and balance training. Furthermore, there is uncertainty about the effect of strengthening exercises alone (without functional exercises or balance training) [28]. This goes with the findings of a previous study that administered resistance exercise using Thera bands in which there was no significant change in both BBS and TUG scores between the groups, or between pre- and post-test [15]. However, another study that also used elastic band resistance exercise reported significant improvement in balance in elderly people [29]. The influence of resistance training on the balance performance of older adults was also investigated by Lee and Park who found that resistance training not only improved strength but also enhanced balance in older adults. They further acknowledged that an improved strength in the lower limb may result in enhancement of balance in older adults who do not have neurological defects. On the other hand, a previous study suggested that the muscle strength gained by resistance training in older adults may not last over time [30]. These variations in results may be associated with methodological differences across the studies such as diverse measurement techniques used, activity level, and other characteristics of the study participants.

From our perspective, balance in older adults can be improved when resistance exercises are used in conjunction with other forms of exercise training like functional-task training or a specific balance training program. Resistance training alone without a balance component may not be helpful in generating positive effects on balance. We propose further research, investigating a combined effect of functional-task training along with resistance training on balance in older people. Future studies utilizing additional measurements methods such as force platforms are suggested to provide support concerning the training programs that contributed to the significant improvement in balance and functional outcomes.

Study limitations

Our study has some inevitable limitations: (1) due to a lack of post-trial follow-up, long-term effects of the training programs were not established; and (2) combined effect of functional-task training and resistance training on balance was also not established.

## Conclusions

The general conclusion to be drawn from this study is that both the training programs were effective in balance enhancement in older adults. However, the balance performance in the FTT group was better than that of the RT group at the end of the training program. As the balance enhancement requires specific, task-oriented balance training, incorporation of functional-task training in exercise interventions would be more beneficial rather than prescribing resistance exercises alone.

## Appendices

Functional-task training program

It is an evidence-based program consisting of exercises that are associated with functional performance. Each of the below exercises was performed for one minute. The complexity and variability of the exercises were progressively increased, as participants gradually became familiar with them. Progression of an exercise included an increase in weight (of the box, tray, ball, etc.), duration, or speed of exercise. The maximum intensity used was "5," i.e. "severe" level of exertion on Borg CR10.

Ø Step on and off a bench.

Ø Sit to stand from a chair while holding an object.

Ø Stopping on commands while walking.

Ø Walking through a curved path.

Ø Walking and turning right/left/back.

Ø Pick an object from the floor and place it on a shelf.

Ø Walking while passing an object from hand to hand.

Ø Walking and avoiding obstacles while listening to music.

Ø Walking up and down a ramp while carrying a weighted box.

Ø Climbing up and down stairs while carrying a weighted box.

Ø Walking and avoiding obstacles while carrying a glass of water on a tray.

Ø Walking and passing through a raised surface carrying a glass of water on a tray.

Resistance training program

The program mainly focuses on lower extremity muscle performance. Each of the below exercises was performed in three sets of eight repetitions. The minimum weight used was 0.5 kg where applicable. Progressions included either an increase in weight or a repetition of an exercise. The maximum intensity used was "5," i.e. "severe" level of exertion on Borg CR10.

Ø Four-way straight leg raises; ankle weights were used to offer resistance.

Ø Seated knee extension exercises: the exercises were performed on a quadriceps table.

Ø Raising the body as high as possible on the toes to strengthen plantar flexors.

Ø Wall slides; introduced additional weight to bodyweight by holding a dumbbell close to the chest with both hands. Alternatively, dumbbells were held in each hand at the sides or over the tops of shoulders flexing elbows.

Ø Step up exercises - forward and lateral; introduces additional weight to bodyweight by holding dumbbells in each hand at the sides or over the tops of shoulders flexing elbows.
